# Speech acts and the communicative functions of emojis in LIHKG online discussion forum amid COVID-19

**DOI:** 10.3389/fpsyg.2023.1207302

**Published:** 2023-07-11

**Authors:** Carol Yu, Dennis Tay, Ying Jin, Xinhua Yuan

**Affiliations:** Department of English and Communication, The Hong Kong Polytechnic University, Kowloon, Hong Kong SAR, China

**Keywords:** speech acts, emojis, computer-mediated communication, online discussion forum, self-help, COVID-19, LIHKG forum

## Abstract

Since the beginning of 2022, the Hong Kong government has imposed strict social distancing measures and changed its stance on various regional policies with the aim to contain the so-called ‘fifth wave’ of COVID-19. In these pandemic and ‘infodemic’ times filled with uncertainty and fear, Hong Kong netizens used local online discussion forums as a resource to establish an innovative form of ‘helping network.’ This study is based on 230 posts from a popular local online discussion forum ‘LIHKG’ in February 2022 when the pandemic was regarded as most critical by the Department of Health. Speech Acts theoretic approach was adopted to explore how forum users employed speech acts to perform various communicative practices such as expressing concerns, asking for information, and engaging with others in a CMC environment amid a global health crisis. Representatives were found to be the most dominant text-based speech acts, followed by directives, expressives and commissives. Speech acts provide forum users a context in which emoji usage occurs. Forum users not only make use of words to ‘do’ things in the online self-help forum, but they also employ emojis to either supplement or complement speech acts. This study also shows that emojis perform multiple functions in the discussion posts and argues that they do not merely function as emotion indicators of their textual company, but also carry significant pragmatic meanings by illustrating how they can also carry illocutionary force and in some cases, even alter the illocutionary force of their preceding texts. The findings of this study enhance our understanding of how forum users communicate via verbal and nonverbal means within the underexplored ‘helping domain’ of online discussion forums. It also suggests that online discussion forum interactions need to be approached differently than other better understood alternatives.

## 1. Introduction

Computer-mediated self-help forums have become increasingly common over the last two decades due to easier access to the internet. Users of self-help forums tend to seek information, advice and psychosocial support through computer-mediated communication (CMC) (Malik and Coulson, 2010). Although different CMC channels offer a wide variety of semiotic resources for individuals to construct and convey meanings, most of the communication in online discussion forums occurs via text-based messages. These messages are often accompanied by emojis, which can be regarded as a compensation for the lack of nonverbal communication cues in CMC settings. Taking a pragmatic perspective, this study examines how Hong Kong netizens made use of a local online discussion forum LIHKG as a resource to establish a ‘helping network’ in which they performed various communicative practices such as sharing personal experiences, expressing concerns, providing information, giving advice and establishing social networks amid COVID-19. Speech acts theory was adopted in this study to uncover and explain the different ‘acts’ performed by forum users via texts. We also investigated the communicative functions of emojis in these messages inductively since LIHKG users were observed to use emojis extensively in their message constructions. By analyzing the speech acts and the emoji usage in these messages, this study investigates how LIHKG forum users made use of these semiotic resources to construct their experiences and achieved social functions in a COVID-19-related thread.

Previous studies have identified some of the advantages that online self-help forums can offer: they allow a greater degree of anonymity as compared to offline support groups, which encourages users to express their thoughts and emotions more freely. The anonymous nature of online support groups especially benefits people with stigmatizing illnesses (i.e., AIDS, breast cancer, prostate cancer) as the online environments were perceived by patients as an easier and safer haven for discussing private or potentially taboo topics (Finn, 1999; White and Dorman, 2001; Coursaris and Liu, 2009). The readily available online self-help groups also allow users easier access which minimizes time and location constraints. Online support groups also have the potential to elicit more information and more varied perspectives from a greater number of users who share similar experiences (Wright, 2000; Walther and Boyd, 2002).

Apart from the advantages offered by online self-help groups, researchers have also been interested in different types of self-help mechanisms and social support that occur in online support group exchanges. A number of studies have adopted content analysis to identify the different types of self-help mechanisms in various online support groups (Finn, 1999; Perron, 2002; Haker et al., 2005). Some important functions of online self-help discussion groups include information sharing, emotional support, advice, social connection, and a sense of community (Klemm et al., 2003; Wicks et al., 2013; Pereira et al., 2021). Members can share information about their conditions, treatments and experiences with others as well as sharing practical advice and tips for coping with their conditions (Bender et al., 2011). Moreover, members can offer emotional support and gain validations by expressing their feelings to others who also go through the same issue, on a platform where they feel safe. Establishing social connections and sense of belonging in online-self-help groups can help reduce isolation and loneliness (Utz and Breuer, 2017). In general, information support and emotional support are found to be the most prominent types of social support provided in computer-mediated self-help groups (Winzelberg, 1997; Braithwaite et al., 1999; Loader et al., 2002; Coulson, 2005).

Overall, participation in computer-mediated self-help groups is associated with positive outcomes including enhanced problem-solving skills, better coping with alienation and isolation (Utz and Breuer, 2017), reduced stress levels (King and Moreggi, 1998) and the establishment of social networks (Finn, 1993; Elstad, 1998). Since most of the communication practices in online discussion forums are text-based, this raises a question: How do people achieve these social functions via their words? We believe speech acts analysis can provide an answer to the question.

Speech Acts Theory (SAT) (Austin, 1962; Searle, 1969) is a relevant theoretical perspective and analytical approach in the present study because it helps us understand how members of online self-help groups create meanings through text, which is the primary semiotic resource for meaning construction in online discussion forums. By analyzing the speech acts performed by members, the intents and purposes behind these constructions can be observed. The application of SAT to analyze speech acts in the LIHKG posts can provide insights and explanations on how LIHKG users share information, express their feelings, gain emotional support and establish a sense of community in online self-help groups during a global health crisis.

Speech Acts Theory was first proposed by the philosopher Austin (1962) in order to explain how people do things with words. This influential theory has since been one of the main streams of study within the field of pragmatics. Austin proposed that all utterances contain both contrastive (descriptive statements which can be either true or false) and performative (utterances which realize social action) elements and the action performed by producing an utterance consists of three related acts: (1) Locutions (the acts of saying something); (2) Illocutions (what is done in saying something) and (3) Perlocutions (the effect of an utterance upon hearers). He proposed classifying the many illocutionary speech acts into five major groups, namely verdictives, exercitives, commissives, behabitives and expositives (Austin, 1962, p. 150). Searle (1976) criticized Austin’s classification of speech acts as ‘defective’ (p. 1) by saying ‘Austin advances his five categories very tentatively, more as a basis for discussion than as a set of established results’ (p. 7). His most prevailing criticism is that there is no consistent principle of classification in Austin’s classification.

Searle revised the speech acts classification and claimed that all speech acts fall into five categories: (1) Representative/Assertive: Speech act that expresses speaker’s belief and that commits the speaker to the truth of what is asserted (i.e., words fit the world. Example: Statements); (2) Directive: Speech act that expresses speaker’s wish and making an attempt to get the hearer to do something (i.e., world fits the words. Example: Requests); (3) Commissive: Speech act that expresses speaker’s intention and marking the commitment for the speaker to engage in future action (I.e., world fits the words. Example: Promise); (4) Expressive: Speech act that expresses speaker’s psychological states which has no direction of fit between the world and words (Example: Apologies) and (5) Declaration: Speech act that brings change in (institutional) reality and has bilateral fit between world and words (Example: Baptizing).

A number of studies have applied speech acts analysis in CMC environments. Vásquez (2011) studied complaints on the travel website TripAdvisor and concluded that complaints co-occurred more frequently with advice and recommendations and they were considered mostly indirect in nature. Other studies focused on users’ self-representation in CMC environments. By examining away messages in Instant Messenger (IM), Nastri et al. (2006) found that they were constructed primarily with assertives, followed by expressives and commissives, but seldom with directives. The authors concluded that away messages tended to reflect both informational and entertainment goals. Similarly, Carr et al. (2012) investigated self-presentation in Facebook status messages and found that they were mostly constructed with expressives, followed by assertives. Their findings demonstrated differences in how users expressed themselves in alternate media. Given that text-based speech acts often co-occur with emoticons and emojis in CMC, some studies have investigated the relationship between speech acts and emoticon usage in message construction. Dresner and Herring (2010) examined the pragmatic function of emoticons and argued that the primary function of emoticon was not to convey emotion but to indicate an illocutionary force, which is the intended effect of the utterance. While their study provided a more nuanced understanding of the functions of emoticons, their study was not situated in a particular CMC setting. In light of this, Skovholt et al. (2014) investigated the communicative functions of emoticons in workplace emails by adopting speech act theory and politeness theory. Through identification of speech acts followed by emoticons in workplace emails, they found that emoticons contributed to modifying the propositional content and the illocutionary force of speech acts, which corresponded with Drenser and Herring’s results (2010). More recently, the popularity of emoji use have attracted scholars’ interests. Ge-Stadnyk (2021) examined and compared how social media influencers on Weibo (a Chinese Microblogging site) and Twitter used emoji sequences when engaging in self-presentation. The study identified a variety of text-based speech acts, emoji functions, and functional relations by conducting speech act and pragmatic function analyses and claimed that emoji sequences functioning as ‘emphasis on text’ was most employed in connection with accompanying texts in both Weibo and Twitter data (p. 378). To our best knowledge, studies on speech acts with emoji usage in self-help online discussion forums is sparse. This study expands the current research scope by examining the text-based speech acts and the communicative functions of emoji in an online self-help discussion forum related to COVID-19, with the aim to investigate how Hong Kong forum users framed their COVID-19 experiences, expressed their emotions and seek socioemotional support from others amid a global health crisis.

As mentioned previously, people employ other nonverbal communication cues to compensate for the lack of facial expressions, bodily moments, intonations and gestures in CMC settings (Walther and D’addario, 2001; Wall et al., 2016; Aldunate and González-Ibáñez, 2017; Esposito et al., 2017). Some of the most widely used nonverbal communication cues in CMC are graphic signs that indicate emotional states in the form of emoticons, and pictographs, in the forms of emojis and stickers (Table 1). The term ‘emoticons’ (a blend of ‘emotion’ and icon’) refers to the graphic representation of facial expressions that are often used alongside the text in computer-mediated communication (CMC). Emoticon was first proposed by the computer scientist Scott Fahlman at Carnegie Mellon University, who used a rotated smiley face: -) and the frowny face: -(to signal his messages were intended as a joke (or not) in a computer science discussion forum in 1982 (Krohn, 2004). Since then, a large number of similar signs have been created. Emoticons are produced with ASCII symbols and are often used at the end of a sentence (Sakai, 2013). Emotions are generally perceived by scholars as paralinguistic elements (Lee and Wagner, 2002; Jibril and Abdullah, 2013) that indicate emotional states (Raymond, 1996; Rezabek and Cochenour, 1998; Wolf, 2000; Derks et al., 2008a,b) since nonverbal communication cues such as facial expressions, intonation, gestures and other bodily movements are missing in CMC settings (Kiesler et al., 1984; Sproull and Kiesler, 1986; Krohn, 2004). The use of emoticons, therefore, serves as a compensation for such valuable yet missing non-verbal cues in CMC (Walther and D’addario, 2001; Wall et al., 2016; Aldunate and González-Ibáñez, 2017; Esposito et al., 2017). Research on emoticon functions have shown that they help to clarify intentions in ambiguous messages (Derks et al., 2008a; Thompson et al., 2016) and to accentuate or emphasize textual messages during CMC interactions (Derks et al., 2008b). The overall aim is to improve the efficiency of CMC communication (Dunlap et al., 2016).

In 1999, the Japanese interface designer Shigetaka Kurita and his team released the first set of emojis that contained 176 pictograms for NTT DoCoMo, a Japanese mobile phone operator. The term ‘Emoji’ is of Japanese origin, meaning e (絵, ‘picture’) + moji (文字, ‘character’) (Bai et al., 2019). Unlike emoticons which are produced by ASCII symbols, emojis are pictograms represented as The Universal Coded Character Set (Unicode) and were initially created for the use on Japanese pager, which then grew its popularity in textual messaging worldwide. In terms of content richness, not only emojis can represent more varied facial expressions as compared to conventional emoticons, they can also represent more abstract emotions and concepts, activities, objects such as animals, plants, body parts etc. (Rodrigues et al., 2018). Given that emojis are ‘the most widely used and standardized symbolic language’ (Bai et al., 2019, p. 4), it has attracted much scholarly attention on diverse research topics including use motivation (Kaye et al., 2016; Gibson et al., 2018), the multiple functions of emoji [see Kralj Novak et al. (2015), Cheng (2017), and Jaeger and Ares (2017) for emotional functions and Na’aman et al. (2017) for semantic function], individual (Herring and Dainas, 2018) and cultural (Derks et al., 2008b) diversity on emoji use. For instance, a recent study conducted by Alharbi and Mahzari (2023) investigated the commonly used emojis, their pragmatic functions and possible gender influences on Arabic tweets and they found that repetition patterns and the tendencies of using certain emojis were influenced by gender differences. The authors stressed that emojis are extremely dependent on context and highlighted the importance of context in studying emojis. Taking a computational approach, other studies investigated the sentiment values of the most commonly used emojis. Kralj Novak et al. (2015) analyzed and formalized the sentiment properties of 751 most commonly used emojis in tweets and constructed the Emoji Sentiment Ranking for automated sentiment analysis. Similarly, Was and Hamrick (2021) established norms for common emoji interpretations by studying young adults’ interpretation of 105 common emojis on Apple OS. While these studies offer valuable resources for sentiment analysis and automated annotation, they are only applicable to a specific emoji set (Apple OS emoji) and the interplay between the emojis and their textual company (i.e., how emojis amplify and modify the overall message meaning together with the textual context) is unknown.

Since the 21st century, the use of stickers has grown its popularity on various instant mobile messaging apps/platforms (i.e., LINE, WeChat, WhatsApp, Kakao Talk). The cartoon-like oversized stickers can be presented in static or animated form and they are usually sent separately without needing to be inserted in text messages (Zhou et al., 2017). Lim (2015) commented that the visual richness of stickers can help users express their feelings more explicitly that cannot be articulated with words, thus attaining what he called ‘communicative fluidity’, (p. 2) i.e., smoother and more seamless CMC communication. Wang (2016) also found that stickers can enhance users’ socioemotional experience since they are more elaborate and expressive than emoticons and emojis and suggested that the combination of text and sticker response can achieve higher level of intimacy.

**Table 1:** Examples of emoticon/emoji/sticker.

Emoticon/Emoji/Sticker	Examples
Emoticon	:-) :-( :D \(^_^)/ =_= T_T	Emoji	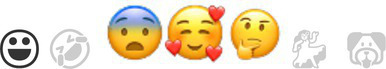
Sticker (Line app)	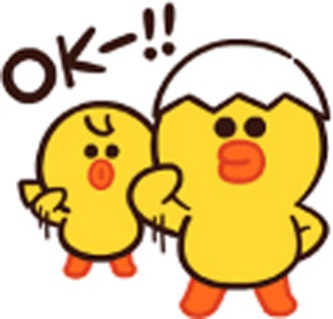 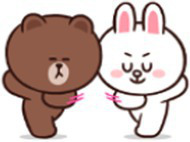 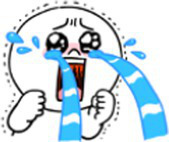 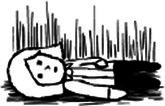

Emoticons, emojis and stickers have been widely used across different platforms and favored in different periods of time. While these expression symbols compensate for the lack of non-verbal cues in CMC environments, their usage is not at all unproblematic. Dresner and Herring (2010) argued that the term ‘emoticon’ is misleading since it implies the main function of emoticon is emotive expression. Their study shows that some typical uses of emoticons are not for emotive indication, but rather indicate the illocutionary force of the text which they are attached to. Considering the pragmatic function of emoticons, they should thus be understood in linguistic instead of extralinguistic terms. Another widely discussed issue with these visual symbols is ambiguity in interpretation. Bich-Carrière (2019) found that the interpretation of emojis can be influenced by users’ cultural backgrounds and technical differences. The user’s understanding of emoji meanings may also differ from their official definitions, causing misunderstanding in interpretation between users (Miller et al., 2016). Lim (2015) also pointed out the interpretation issue with stickers, claiming that ‘the interpretability of the stickers also lent our messages an air of equivocation, allowing the conversation to be shaped by the different parties as it went along’ (p.3). The different understandings and interpretations of these visual symbols can cause inefficiency in CMC communication, it may even lead to discourse interruption and cause damage to interpersonal relationships (Tigwell and Flatla, 2016).

Speech acts provide forum users with a context in which emoji usage occurs, i.e., forum users make use of speech acts to ‘do’ things in the LIHKG self-help forum using texts, while also employing emojis to either supplement or complement speech acts. This study aims to investigate Hong Kong netizens’ communication practices in a specific discussion thread related COVID-19 on a local discussion forum LIHKG by (1) identifying the text-based speech acts in the discussion posts and (2) analyzing the functions of emojis and how they relate to their accompanying texts.

## 2. Materials and methods

### 2.1. Research context

Established in 2016, LIHKG is a popular online discussion forum in Hong Kong. Before its rise in popularity, HKGolden Forum used to be the main online discussion forum in the local community. However, due to user restrictions and censorship issues, a growing number of users left HKGolden Forum and shifted toward LIHKG for a better user experience. Soon after its establishment in 2016, LIHKG attracted 70,000 registered users with around 1,400 posts and 70,000 replies per day (Gap, 2016). In 2019, LIHKG was voted the most critical medium by over half of the respondents in a newspaper poll conducted during the Anti-Extradition Law Amendment Bill (Anti-ELAB) demonstration on July 1, 2019 (Jacobs et al., 2022). The anonymous nature afforded by LIHKG was perceived by activists and netizens as vital, making it a preferred medium for expressing their political views (Erni and Zhang, 2022). According to Similarweb (2023), which offers statistical data for top websites, LIHKG is currently the third most popular social media site (as of March 2023) in Hong Kong, with over 25 million visits per month.

LIHKG offers 41 discussion channels that cover a range of topics, including social affairs, housing, finance, academics, health and love affairs, to name a few. Visitors do not need to register for an LIHKG account to read open posts, but they do need a registered account for creating threads, leaving comments and reading encrypted posts. In order to be registered and verified by LIHKG, users have to register with an accredited ISP email address or by one of the UGC-funded Hong Kong universities email address.

**Figure 1:**
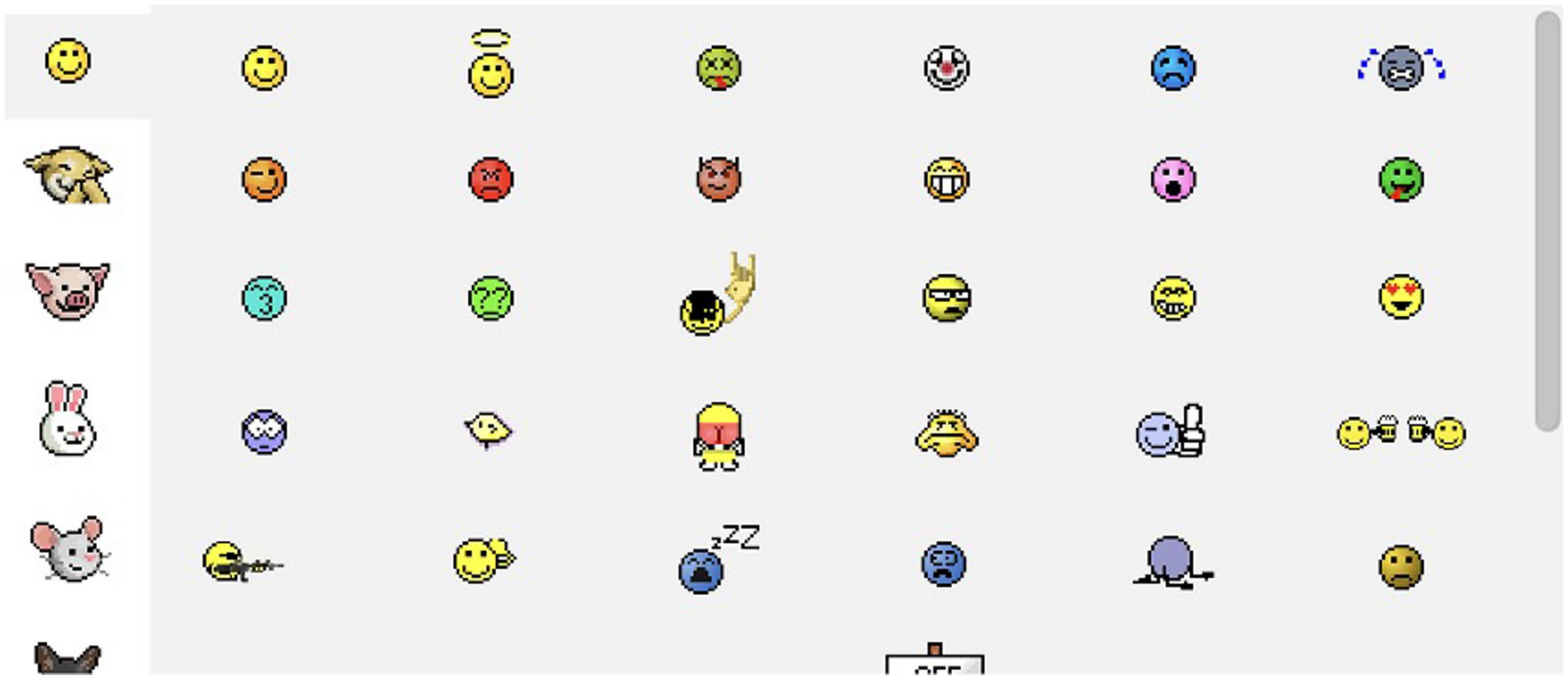
Examples of LIHKG emojis.

**Figure 2:**
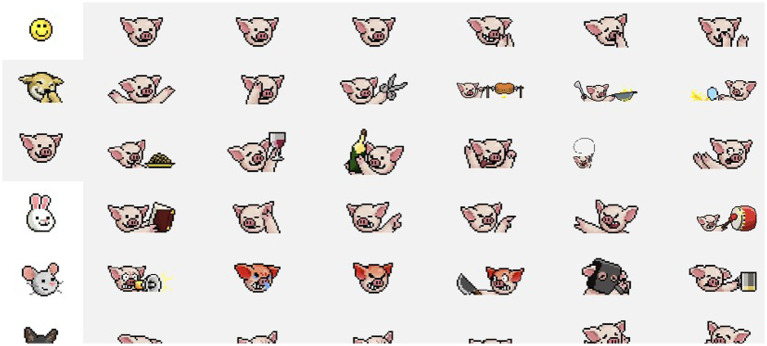
LIHKG Pig emojis.

There are approximately 440 customed emojis (static and animated) offered by the LIHKG discussion platform as of March 2023. This number is not definite as LIHKG releases different emoji sets on many different occasions (for instance, emojis for Chinese New Year, Christmas and World Cup themed emojis). Apart from the more standardized emojis that represent facial expressions and emotions (Figure 1), many LIHKG emojis are found to represent actions and bodily movements which are mainly organized by animals. The most famous animal-mascot emojis on LIHKG are the LIHKG Pig (連豬; lin zyu) (Figure 2) and LIHKG Dog (連狗; lin gau). Which were made protest figures/mascots during the Anti-ELAB movement in 2019 (Jacobs et al., 2022).

### 2.2. Data collection

Since the Hong Kong government’s announcement of the so-called ‘Fifth-Wave of COVID’ in the city at the beginning of 2022, there had been a sharp increase of positive Covid cases recorded from the 26th February 2022 and reached its peak on the 1st March 2022 (Centre for Health Protection, 2023). The fifth-wave wave of COVID-19 struck Hong Kong really hard in terms of velocity, infection and death cases as compared to the previous four waves (Table 2). In order to contain the ‘Fifth-Wave’ of COVID-19, the Hong Kong government imposed strict social distancing measures and changed its stance on various regional policies which inevitably affected Hong Kong citizens’ way of life. In times filled with uncertainty and fear, Hong Kong netizens used LIHKG discussion forum as a resource to seek health information, to express their emotions toward the government and its policies and to establish an innovative form of ‘helping network’ among other LIHKG users.

**Table 2:** Total number of reported and death cases in different waves of COVID-19 in Hong Kong.

Waves of COVID-19	Period	Total number of reported cases (by nucleic acid tests and rapid antigen tests)	Death cases (Fatality rate)
1st	23 January 2020 to 14 March 2020	142	4 (2.8%)
2nd	15 March 2020 to 30 June 2020	1,064	4 (0.38%)
3rd	1 July 2020 to 31 October 2020	4,118	103 (2.5%)
4th	1 November 2020 to 30 April 2021	6,451	101 (1.6%)
5th	31 December 2021 to 29 January 2023	2,863,475	13,120 (0.46%)

Data adapted from Wong et al. (2022) and Centre for Health Protection of the Department of Health and the Hospital Authority (2023).

A specific thread titled ‘RAT +ve/初步確診/確診圍爐區’ (Rapid Antigen Tests (RAT) + ve/ Preliminary Confirmed/Confirmed support group) has been dedicated to RAT self-testing on LIHKG forum. This thread first appeared on the 14th February 2022, during the fifth-wave of COVID-19 in the city and contains 154 pages (as of 21 Mar 2023) with each page containing 1,001 posts/comments. Altogether, there are over 150,000 posts/comments under this thread since it was created. The data collected in the present study followed The Robots Exclusion Protocol which instructs search engines whether or not a page can be indexed, archived or summarized (LIHKG’s Robots agreement^1^). The data in the present study comprises a total of 230 both open and encrypted entries posted under this specific thread on the 27th February 2022, when the number of infections were rocketing. The average length of posts is 28.9 words (SD = 129.4). The original posts were written in colloquial Cantonese, the language spoken in Hong Kong, and were translated into English for the present study. Permission to illustrate LIHKG emojis in this article has been granted by LIHKG.com. The nature of support offered by this ‘helping network’ is also reflected in the title of the thread “RAT +ve/初步確診/確診圍爐區’ (Rapid Antigen Tests (RAT) + ve/ Preliminary Confirmed/Confirmed support group).

### 2.3. Analytical procedures

The analysis consisted of two steps: (1) identification of text-based speech acts in 230 continuous posts under the same discussion thread and (2) interpretation of emoji functions with their accompanying texts. We adopted Searle’s speech acts taxonomies (1969) in the speech acts identification process. They include Representative/Assertive, Directive, Commissive, Expressive, and Declaration. During communication, speakers/writers may use multiple clauses to perform the same illocutionary act. In this study, we concur with Garcia’s (2004) claim that ‘a unit of analysis that takes illocutionary meaning into account, beyond solely grammatical or intonational boundaries, was deemed most appropriate’ (p. 52) and adopted speech act as the basic unit of analysis. We then analyzed the communicative functions of the emojis inductively and interpreted them alongside the speech acts they accompany. Since speech acts and emoji are highly context-dependent, identifying and interpreting them require researchers’ close reading of the texts and their contextual environments. To ensure consistency, the first author, who is a native Cantonese speaker, compared and rechecked the coding and interpretations periodically along the analytical processes. To increase reliability, the data was coded independently by the first and third authors. Peer checking was also carried out after the identification and interpretation processes. Percentage agreement between the two coders on speech acts identification was 79.6%. Continuous discussions were carried out among all authors to resolve disagreements until consensus was reached and agreed upon.

## 3. Results

### 3.1. Speech acts identification and distribution

A total of 262 speech acts were found in our data of 230 posts within the same discussion thread. A post may contain zero (no text, only emoji) to multiple speech acts (user can share personal experience, express emotion and ask for advice in the same post). Table 3 summarizes the speech act distributions in our data (Table 3).

**Table 3:** Speech acts distributions.

Speech acts	*N*	Standardized residuals
Representative	156 (59.5%)	+14.31
Directive	69 (26.3%)	+2.29
Expressive	30 (11.5%)	−3.09
Commissive	7 (2.7%)	−6.27
Declaration	0	−7.24

Representatives was found to be the most dominant speech act (59.5%, *N* = 156), followed by directives (26.3%, *N* = 69), expressives (11.5%, *N* = 30), and commissives (2.7%, *N* = 7). No declaration was found. A Chi-Square Goodness of Fit Test was performed to determine whether the speech acts were equally distributed among the five categories. The results [*X^2^*(4, *N* = 262) = 311.4, *p* = 0.0001] show significant differences in the distribution between all five categories (see Table 3), with each category occurring significantly more common than the next. In order to gain a better understanding of such speech act distributions, further identification of each speech act type was carried out, respectively.

#### 3.1.1. Representatives

Representatives are speech acts that express speaker’s belief and that commit the speaker to the truth of what is asserted (Searle, 1969). By employing representatives, LIHKG users represented the world as they believe to be the case (or not). Representatives comprised of five speech acts in our data: Sharing personal experience, sharing personal opinion/belief, providing information, joking and correcting. Table 4 shows the number of counts, percentage and example for each act. Sharing personal experience was found to be the most prominent speech act under representatives. By sharing their experiences during the COVID-19 ‘fifth-wave’ on LIHKG forum, users could gain support and empathy from each other who went through similar situations (Post 54). Moreover, sharing personal experience was found to occur with requesting information (directives) in a number of posts and functioned as providing contextual information that foregrounded a request. As shown in Post 46, the user detailed his/her grandparents’ infected situations via representatives before asking for opinions (directives).

**Table:** 

Post 46 (Original post)	Post 46 (Translation)
阿剬阿婆兩個都80歲以上，冇長期病患 今日快測發現中咗 阿婆乜事都冇，阿剬見感冒，兼且撞聾 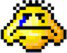 結果唔係好溝通到，淨係知佢好似唔嚴重 而家叫佢食住panadol先，叫阿婆睇住佢，如果好唔妥就直接999 目前係咪咁處理係最好 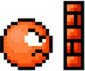	My grandparents are both over 80 without any chronic illnesses. They were tested positive today. My grandmother is fine but my grandfather has flu symptoms, and has hearing problem 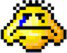 So I cannot really communication with him. I only know he does not seem to be seriously ill. I told him to take Panadol for now and asked my grandmother to take care of him. Will call 999 [emergency hotline] if he falls very sick Is this the best way to handle the situation for now? 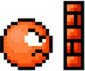

**Table 4:** Representative speech acts.

Representatives		*N*	%
Sharing personal experience	Post 54:隔離咗10日都仲positive 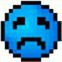 (*Still positive after 10 days quarantine* 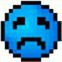 )	68	43.6%
Sharing personal opinion/belief	Post 170: 玩完 你成條T線直沖出黎 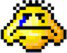 應該準備發燒 *[It is over. The whole ‘T’ line is showing* 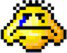 *(You) ready to have fever.]*	50	32.1%
Providing information	Post 53:快測冇amplification核酸少少病毒都度到 *(There is no amplification in RAT test. Even tiny amount of virus can be detected with PCR test.)*	31	19.9%
Joking	Post 56: 有得放長假 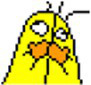 (*Can have long vacation* 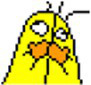 )	5	3.2%
Correcting	Post 137:係喉嚨呀 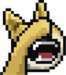 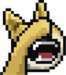 *(It is throat* 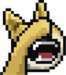 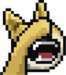 ) [typo correction]	2	1.2%

Apart from sharing their personal experiences, users were also found to share their opinions and beliefs toward the COVID-19 symptoms, RAT test results (Post 170) and government policies. As this LIHKG thread was a convenient and popular site for users to exchange information about COVID-19, providing information was also a common speech act (Post 53), accounting for almost 20% of representatives.

#### 3.1.2. Directives

Directives are speech acts that speakers use in order to get the hearers to do something (Searle, 1969). Directives found in our data can be categorized as: requesting information/opinion, giving advice, giving order/command, wishing and demanding. As Table 5 shows, requesting information/opinion makes up the majority of directives, suggesting that users made use of LIHKG forum to obtain COVID-19-related information was a common practice (Post 9). Not only users used directives for requests, they also used them as a means to give advice and suggestions to other users (Posts 109 and 133).

**Table 5:** Directive speech acts.

Directives		*N*	%
Requesting info/opinion	Post 9: 收到初步確診訊息，但係冇收到手帶，咁算唔算隔離人士 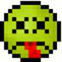 *(Received preliminary confirmed diagnosis but have not received the wristband. Am I regarded as a quarantine case* 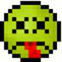 *)*	53	76.8%
Giving advice	Post 109: 拎醫生紙先啦 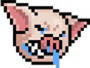 *(Get medical certificate first* 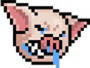 *)* Post 133: 冇病徵都休息多啲 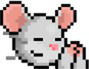 *(Take more rest even if you do not have symptoms* 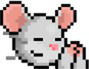 *)*	8	11.6%
Giving order/command	Post 145: 得咁就唔好放出黎啦 冇哂食慾 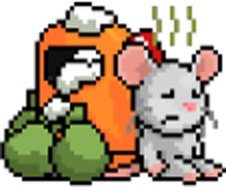 *(Do not post it here if that’s all you have got. I’ve lost my appetite* 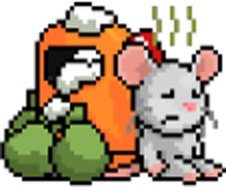 ) [*In response to another user who posted his half-naked picture*]	5	7.2%
Wishing	Post 42: 唔好中其他野啦 *(Do not get infected with other things)*	2	2.8%
Demanding	Post 122: 清唔好嚇我 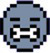 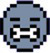 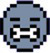 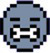 (*Brother do not scare me* 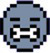 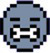 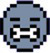 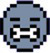 )	1	1.4%

While advice can sometimes appear in imperatives (e.g., Post 109), which is conventionally used in acts of command and order, there is a fundamental difference between advice and command. Searle (1969) stated that giving advice is a speech act that the speaker believes what he/she says will benefit the hearer and according to Brown and Levinson (1987), advice is to tell what is best for someone. In this sense, giving advice is considered to be beneficial to the hearer, rather than the speaker. However, giving advice is also regarded as a potentially face-threatening act (FTA) (Brown and Levinson, 1987) since it places the hearers in the position of doing something that has been advised, thus limiting the freedom of the hearer. Therefore, Hinkle (1997) warned that giving advice must be performed with caution and the speech act of advice should be softened so as to not offend the hearer. This may explain why writer of Post 109 made use of the crying LIHKG pig emoji to soften the speech act of advice (more detailed discussion of emoji functioning as a marker to attend to the addressee’s face needs in Section 3.2.5).

Despite its low frequency, giving order/command was also observed as one of the directive speech acts in our data. Interestingly, they were only found in chit-chat, i.e., discussion topics that deviated and had nothing to do with COVID-19 and RAT test. Examples of such deviated topics included food preferences, physique, showering habits and sexual topics (see Post 145 as an example). A possible explanation of such a phenomenon is that the act of giving order/command is inherently face-threatening (Brown and Levinson, 1987) and they might not have been taken as seriously in more light-hearted discussion topics such as the ones stated above as compared to more serious topics related to COVID-19. Moreover, giving commands requires the preparatory condition that the speaker having some kind of authority over the hearer (Searle, 1969). Given the anonymous nature of LIHKG forum, such information was not available to the users. So essentially, no one would be regarded as having the authority nor the legitimacy to give order and command on medical topics to other users.

#### 3.1.3. Expressives

Expressives speech acts are acts that express the psychological states of the speakers (Searle, 1969). Speakers use them to express how they feel. In our data, expressives include the following speech acts: expressing emotional/psychological state, expressing desire, complaint, sarcasm, appraisal and greeting. Table 6 illustrates that expressing emotional state takes up the majority of expressives (60%). They were typically used to state how the users felt with issues related to COVID-19 (Posts 59 and 122). Users also used expressives to express their desires, as seen in Post 128 in which the writer expressed his/her desire to get out of the house during the quarantine. Complaint was also identified as expressives as it helped the writer to state their discontent and dissatisfaction toward someone/something. Post 182 illustrates the resentment of the user toward the Department of Health and their confusing quarantine policies.

**Table 6:** Expressive speech acts.

Expressives		*N*	%
Expressing emotional/psychological state	Post 59: 好慘 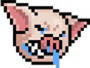 *(So pitiful) 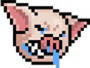 [in response to another user who stated he/she did not take a shower due to infection]* Post 122: 我屋企人中招已經好撚心慌 佢又無打針 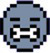 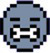 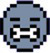 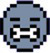 (*My family member is infected and that made me so scared. He/she is not vaccinated* 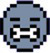 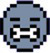 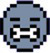 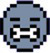 )	18	60%
Expressing desire	Post 128: 好想出街 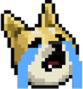 (*Really want to go out* 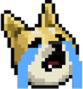 )	7	23.3%
Complaint	Post 182: 真係吾知佢地做乜撚野 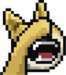 (*Really do not know what the hell they [Department of Health] are doing* 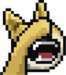 )	2	6.7%
Sarcasm	Post 160: 歡迎加入 *(Welcome to the club [as confirmed COVID case])*	1	3.3%
Appraisal	Post 184: 正 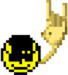 (*Cool* 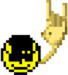 ) [Appraised the loosened quarantine measures]	1	3.3%
Greeting	Post 201: 康文巴 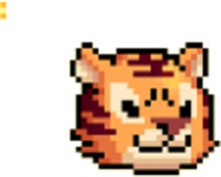 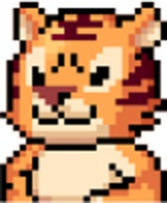 *([another LIHKG user ID]* 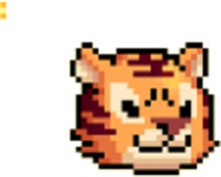 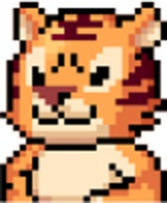 )	1	3.3%

#### 3.1.4. Commissives

Speakers use commissives to state their intends. In other words, they are used to state speakers’ commitments to future action. Only seven commissives were found in our data and they all signaled users’ intentions to commit to some future actions (Posts 137 and 142) (Table 7).

**Table 7:** Commissive speech acts.

Commissives		*N*	%
Committing to future action	Post 137: 可以買定喉糖 *([I] can buy some throat lozenge in advance)* Post 142: 都係測多幾次隱陣啲 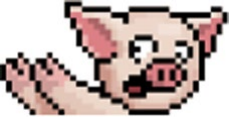 *([I am] Going to take a few more [RAT] tests just to be sure* 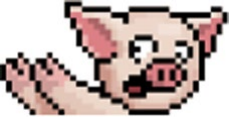 )	7	100%

#### 3.1.5. Declarations

No declaration was found in our data and, given the function of this speech act, this is not surprising. In order to perform declaratives, speakers need to have some kind of institutional or authoritative role in a specific context so that his/her utterances can induce change in the world/reality. LIHKG as an online discussion platform does not have such institutional power. Furthermore, as mentioned previously, the anonymous identity of LIHKG users prohibits the exhibition of institutional roles, thus restraining the legitimation of performing declarative speech acts.

### 3.2. Communicative functions of LIHKG emojis

A total of 290 emojis were found in 173 posts in our data. Fifty seven posts (24.8%) were found to contain no emoji. For posts that employed emojis, users made use of as little as one to as many as 18 emojis within a post. The heavy use of emojis suggests that they are an integral element for meaning construal in LIHKG forums. This section accounts for their typical communicative functions in the discussion thread.

#### 3.2.1. Emphasizing textual content

This type of emoji represents the propositional content conveyed by the text in a message and their use are dependent on their textual environments. They emphasize textual content by repeating it (Ge-Stadnyk, 2021). A direct mapping of textual meaning and graphical signs can be deduced. They do not contribute, modulate nor alter the propositional meaning of the texts. In our opinion, they serve as a graphical representation of the textual message with the aim to emphasize textual content and potentially enhance the visually attractiveness of the message. An example of this use is illustrated in Post 211. The user made an evaluative comment on the latest quarantine measures released by the Hong Kong government in February 2022, criticizing them as illogical. The laughing LIHKG dog that appears in the beginning of the message mimics the word ‘laugh’ in the phrase ‘I fucking laugh’ that follows.

**Table:** 

Post 211: 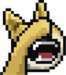 笑撚咗 其實幾冇logic	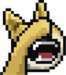 *[I] fucking laugh. This is in fact illogical*

#### 3.2.2. Intensification

Emojis can also be used to intensify propositional content and modulate the intensity of an already identifiable act (Dresner and Herring, 2010). In response to an earlier message posted by another LIHKG user who claimed that he/she had not taken a shower for a day due to infection, the writer of Post 59 made an expressive speech act ‘so pitiful’ ‘好慘’ to express his/her sympathy toward the person. The crying LIHKG pig emoji in this post can be interpreted as intensifying the affective value expressed in its textual counterpart and altogether, the whole message containing both text and emoji helped the writer express his/her sympathy toward the other user.

**Table:** 

Post 59: 好慘 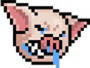	*So pitiful* 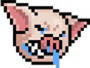

#### 3.2.3. Marker of negative attitudes

One of the main functions of emojis found in our data is that they acted as contextualization cues (Gumperz, 1982) by providing extra information to help readers understand and interpret the intended meanings expressed in the texts. More specifically, our findings show that LIHKG users often employed emojis to express negative attitudes which were not explicitly stated in the texts when framing their COVID-19 experiences, as shown in posts 72 and 92 below:

**Table:** 

Post 72: 墳緊張申報form 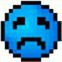 好驚入亞博	*Filling in declaration form [for RAT + ve]* 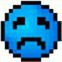 *I’m scared that I may need to get into Asia-Expo [quarantine venue]*

The representative statement of ‘Filling in the declaration form’ does not actually contain any affective elements. The negative emotion is only made explicit with the frowning emoji 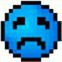 that follows, which frames the act of form-filling as a saddening procedure. This negative emotion is then confirmed by the expressive act ‘I’m scared that I may need to get into Asia-Expo’ that comes after. Without the frowning emoji, the readers might have interpreted the writer as being scared only. The use of frowning emoji here can be seen as providing cues to the readers by making the implicitly implied negative emotion explicit. Thus helped them interpret the whole event as not only a scary but also a saddening one. Similar usage can also be observed in the example below. The writer made a hypothetical commissive act via words ‘I’m going to ignore it if I do not have any symptoms, even if I got tested positive 7 days in a row’ without stating his emotion and psychological state explicitly. His/her negative attitude can only be inferred in the second phrase ‘Need to make a living’. The writer made use of the crying 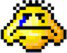 emoji after the first phrase and the frowning 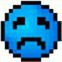 emoji after the second phrase to help him/her express negative attitudes and framing the event as a negative one.

**Table:** 

Post 92: 如果7日都仲係陽 冇病徵想唔理算 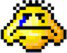 要搵食呀大佬 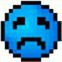	*I’m going to ignore it if I do not have any symptoms, even if I got tested positive 7 days in a row* 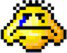 *Need to make a living* 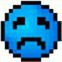

The above examples show how emojis function as negative attitude markers that complement the implicit affective meanings made in texts explicitly. In some other cases, no affective meanings nor implicit affective attitudes can be found in the texts and emojis in such cases serve as independent expressive act that complete the overall meaning of the messages, providing clues to readers as to how they should interpret and understand the overall meaning of the messages. The writer of Post 159 responded to a previous post that requested information on sick leave application procedure since he/she was confused by the boss’s ambiguous reaction toward his/her infection. Writer of post 159 then responded with directive acts (requesting information and giving advice), followed by a representative act of sharing his/her own experience:

**Table:** 

Post 159: 你收到sms確診未 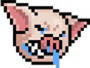 收到就book診所先 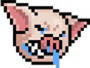 我嗰時都冇同我講係sl定乜 我自己係屋企等衛生署call 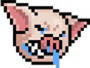 後尾覺得唔撚對路都係去診所拎醫生紙 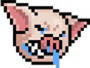 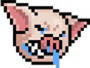 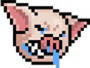	*Have you received the sms confirmation message* 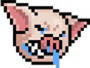 *If you have, then book a clinic first* 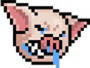 *They also did not tell me whether I got any sl [sick leave] or whatever. I waited at home for Department of Health’s phone call* 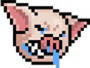 *. Then I thought something was not right so I went to the clinic and got a medical certificate* 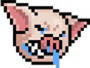 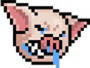

The writer did not express any of his/her emotion through the texts. However, this was achieved through the use of multiple crying pig emojis. These emojis then function as independent expressive act that served the writer’s intention of framing his/her experience as a negative one through negative emotion expression. Together with the directive and expressive acts realiszd via verbal means, the expressive act carried out by the emojis completed the meanings intended by the writer.

#### 3.2.4. Marker of sarcasm

Emojis can also function as marker of sarcasm. In Post 233, the writer raised a question about quarantine policies:

**Table:** 

Post 233: 如果冇嘅密切接觸者要14日 但確診者7日? 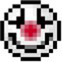 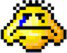	*If no close contact [with infected person] then 14 days [quarantine] But confirmed cases 7 days [quarantine]?* 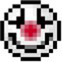 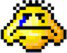

He/she first pointed out the quarantine policy using representative act, which was then followed by a directive (question – requesting an answer). This message should not be taken literally as a question though as this was hinted by the use of the clown smiley emoji 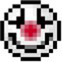. This emoji is conventionally known as a ridicule on the LIHKG platform and is usually used to signal something or someone as nonsensical and ridiculous. By using this emoji, the writer implied that the quarantine policy was ridiculous instead of genuinely asking for an answer. This smiley thus conveyed the writer’s epistemological stance in the utterance by framing the question with a sarcastic note which turned it into an assertion of writer’s opinion. As a result, the pragmatic meaning and the illocutionary force of this utterance were altered by the insertion of the clown smiley emoji 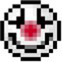. After giving out this interpretation clue to the readers, the writer then used a crying emoji that expressed his/her sadness for the need to comply to the policy even though it was deemed ridiculous to him/her.

#### 3.2.5. Marker to attend to addressee’s face needs

Some emojis were used to attend to the readers’ face need. The notion of ‘face’ (Goffman, 1967) is situated within the frame of politeness theory (Brown and Levinson, 1987) and refers to a person’s public self-image when participating in interaction. During social interactions, people generally expect their public self-image, or their face wants, to be respected. ‘Face’ is further categorized as (1) negative face: the need to be independent and not to be imposed by others and (2) positive face: the need to be accepted and approved of. Examples below illustrate how emojis attend to readers’ face needs through mitigating the illocutionary force of face-threatening acts (FTAs):

Post 65 is a reply to a previous post that offered suggestions on medicine and grocery stocking. The writer asserted that he/she had enough of both and told his/her interlocutor not to worry, with an animated kneeling and bowing emoji 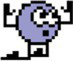
at the end of the sentence. This emoji performs multiple functions in this post. On one hand, it expressed the writer’s gratitude via the expressive act of thanking which was not expressed in the verbal means. Therefore, functions as a contextualization cue to the reader to interpret the message as an expression of thanks which oriented to the addressee’s positive face need of being appreciated. On the other hand, it softens the illocutionary force of its preceding directive ‘No need to worry’ which could have been interpreted as an FTA of command. This emoji thus serves as a face-threatening mitigation device that directed to addressee’s negative face want.

**Table:** 

Post 65: 藥同食物都好夠，呢樣唔駛擔心 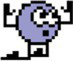	*Medicine and food are sufficient. No need to worry* 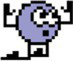

This emoji usage can also be found in the example below:

**Table:** 

Post215: 有冇人知道打咗兩針但確診咗之後係咪未有延遲打第三針嘅安排?想要source 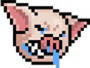	*Does anyone know if the third injection arrangement would be delayed after receiving two injections but infected? Want source* 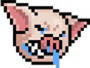

The crying LIHKG pig emoji in the example above not only functions as an expressive act of writer’s affective state but also serves as a FTA mitigation device to soften the force of requesting information source.

In some cases, the FTA is so overt that without any mitigation devices, the message would have been taken as offensive.

**Table:** 

Post103: 屌你 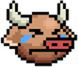 呢度冇人填左?	*Fuck you* 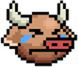 *Has anyone here filled it [RAT + ve declaration form]?*

After stating the fact that he/she was filling in the RAT test +ve declaration form and that he/she was worried to be quarantined in an earlier post, another user made a sarcastic reply and told him/her to be ready for quarantine. The writer in post 103 then replied with an expressive act ‘Fuck you’ to express his/her discontent toward that reply. In fact, swearing is not uncommon on LIHKG discussion platforms and social swearing can be regarded as a social cohesive device signaling group membership (Montagu, 2001) within the LIHKG community. Nevertheless, the writer opted for inserting a crying LIHKG cow emoji as an expressive act to (1) express his/her affective state when facing the uncertainties and worries and (2) mitigating the overt FTA and soften its illocutionary force by inviting and eliciting empathy using the crying emoji.

#### 3.2.6. Approximation strategy device

Within the theory of communication accommodation (Giles and Ogay, 2007), approximation strategy is concerned with communication production via adjusting one’s speech to be more like his/her interlocutor through any salient communication features such as accent, speech rate, word choices and other nonverbal behaviours that aims to gain social liking and approval (Gallois et al., 2005). The strategy, stemming from Similarity-Attraction Paradigm (Byrne, 1971), predicts that similarity on attributes such as attitudes, values and beliefs can facilitate interpersonal attraction. Approximation strategy posits that one person’s speech style becomes more similar to the other during interactions which increases social liking from one’s interlocutor. In a similar vein, emojis also serve as nonverbal approximation device in CMC settings. Users can make use of the same emojis in replies to make their messages more ‘similar’ to the ones of their interactants:

**Table:** 

Post 20:14日好撚爽 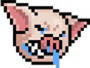 出嚟做嘢冇放過咁耐 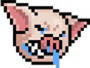 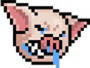 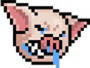 Post26: 屌你我都唔知有冇14日 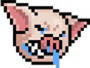 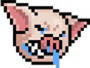 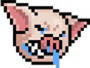 酒店有未 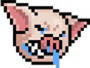 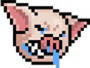	*Post 20: 14 days [of sick leave] is so fucking cool* 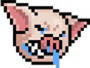 *Never have had such long holiday* 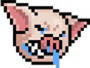 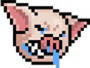 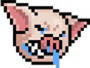 *Post 26:Fuck you I do not know if I would have 14 days* 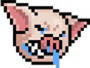 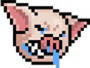 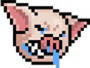 . *Are the [quarantine] hotels available yet* 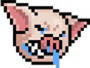 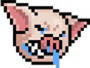

The example above shows how interactants made use of the same crying LIHKG pig emojis over the exchange. There are in fact a variety of crying emojis available on LIHKG so the fact that the respondent chose to use the same emoji in his/her reply may be interpreted as an approximation tactic achieved by collective effort that aimed for rapport building.

## 4. Discussion

As suggested by group work theory (Rose, 1977), a well-functioning group should be able to satisfy both the task and socioemotional needs of its members. The findings of the current study show that this specific LIHKG discussion thread provided a venue for its users not only limited to COVID-19 information exchange, but also socioemotional expressions which supports previous studies that investigated online group functions (Finn, 1999; Malik and Coulson, 2010).

In this study, we took a pragmatic perspective and adopted speech acts theory as our theoretical approach to analyze the LIHKG posts and their intended meanings through speech acts identification and investigation on the communicative functions of emojis. Our analysis of text-based speech acts in the LIHKG thread shows that representatives dominate in the overall speech acts distribution. LIHKG users mainly made use of representatives to share their personal experience and opinions and to provide information on COVID-19 related issues. This is followed by the use of directives which users used to request information and opinions, give advice, command and order. The third most employed speech act was expressive that helped users express their emotional/psychological states and their desires. In several cases, LIHKG users also used expressives to complain about, to make sarcastic remarks on and to appraise government quarantine policies. Commissive speech acts that indicated LIHKG users future action commitments ranked fourth in the distribution. No declaratives had been found.

Although expressive speech acts ranked third in the overall speech acts distribution, it does not necessarily mean that LIHKG users did not prioritize their emotion and psychological states in the discussion posts. In fact, our analysis of emojis’ communicative functions shows that users made extensive use of emojis as negative attitudinal markers to reveal their emotional and psychological states, with and without their accompany text. This suggests LIHKG users’ preference of employing multiple semiotic resources to express their inner states and explains the reason why text-based expressive speech acts only ranked third in the overall text-based speech acts distribution.

The abundant use of emojis found in our data shows that it is an integral meaning making component for LIHKG users. In general, they serve as contextualization cues (Gumperz, 1982) that provide extra information to readers as to how a message should be understood, interpreted and responded to which is in contrast to Walther and D’Addario (2001) earlier study on emoticons, the precursor of emojis, in which they concluded that ‘emoticons had few impacts on message interpretation’ (p. 341). In the current study, they were employed as attitudinal markers to help users express their emotion and psychological states, which has been well researched and proven to be an important function of emoji usage (Gülşen, 2016; Kaye et al., 2016). Our analysis on emojis’ communicative functions in a specific LIHKG thread shows that they also performed other communicative functions. They can emphasize textual meanings, intensify the propositional content of a message, and even alter the illocutionary force of its preceding texts (as in the case of sarcasm) which supports Dresner and Herring (2010) observation on the illocutionary force of emoticons. They also serve the function of attending to addressees’ face needs. While the same emoji appeared in a string of replying posts, they acted as approximation devices with the aim to gain social connectedness which can enhance group cohesion in CMC which is one of the important functions of online self-help discussion group,

The large number of emojis which signal negative emotional and psychological states in this COVID-19 related thread also confirms that situational factor, i.e., the topic of discussion in our case, appears to influence emoji choices. Previous research has suggested that demographics such as age, gender, cultural backgrounds and individual psychological differences can affect emoji use (Herring, 2007; Alharbi and Mahzari, 2023). However, given that identities is a highly sensitive issue in the LIHKG forum, such information was not available in the current study and thus could not be verified.

During the analysis, we also came across some cases in which emoji usage was ambiguous, making it difficult to determine the rationale behind emoji use (Jaeger and Ares, 2017). For instance, the animated ‘chewing’ emoji in the example below does not support the ideational meaning conveyed by the text, nor does it express a certain emotion/psychological inner state.

**Table:** 

Post 38: 你係sms收到先開始請14定幾時開始 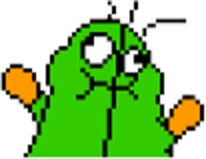	Did you apply 14 [sick leave] after you received the sms 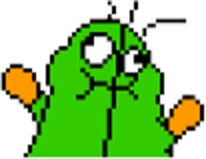

For what reason then, the user chose this particular emoji and incorporated it in this post? This raises a fundamental question on the motivation and interpretation issues of emoji usage. Unlike face-to-face communication in which speaker’s inner thoughts, emotion and psychological state may be ‘given off’ via unintentional facial expressions (Dresner and Herring, 2010), the employment of emojis in CMC is an intentional construal of meaning, but is the choice of emoji always rational and therefore, can be appropriately interpreted? Answering this question would require conducting interviews with the writers to find out the motivations behind emoji usage.

The present study fills the research gap of the lack of meaning construction research in online self-help groups and addresses the issue of how group members realize the information and emotional support functions of online support group via meaning construction in the discussion posts using the multimodal semiotic resources (i.e., text and emojis) afforded by LIHKG. The nuances of speech acts and emoji usage suggest that one needs to consider the multimodal and situated nature of the messages to better understand the content richness in the online discussion posts.

## 5. Limitations and future research

One limitation of the study is that we only analyzed a very small sample size. The identification of speech acts and the highly context-dependent communicative functions of emojis required extensive close reading which inherently limited the data size for qualitative analysis. The speech acts identification of this study are not meant to be representative nor generalizable in other online discussion forums. Likewise, the communicative functions of emojis presented in this study are by no mean exhaustive. Nevertheless, we have presented how LIHKG users employed speech acts to perform ideational-based and socioemotional-based tasks and some typical usage of emojis in the COVID-related discussion thread. With a larger sample and more robust coding scheme, a more quantitative approach could also be taken to identify recurrent discourse form-function pairings in such online discussion forums (Tay, 2015).

Another limitation concerns with the issue of interpretation as discussed earlier. The heavy reliance on the judgment and intuition of the researcher is an inherent limitation of discourse analysis (Powers, 2001). Our analysis oriented to investigate the writers’ communicative intentions and their use of emoji in the exchanges but their motivations are unknown. It would have been ideal to conduct interviews with the writers and ask questions about their motivations and message interpretation to triangulate and validate our findings. However, as LIHKG forum is anonymous in nature, conducting interviews with them may not be feasible. Anonymity also prohitbied us to study how demographic variables may influence emoji use and interpretations within the LIHKG community.

Since exchanges occurring in LIHKG threads are loosely structured and rather spontaneous (Lee, 2020), it would be useful to compare the speech acts use patterns found in this study to a more structured f2f setting to explore if and how people make use of speech acts differently in discussing COVID/health-related issues in different settings. As topic of discussion can influence the choice and patterns of emoji use, future studies may also gain better insights on emojis by investigating their usage and functions in other LIHKG threads. Additionally, it may also be feasible to conduct comparable studies on Western discussion forums with English as medium to explore how cultural and linguistic factors play their roles in speech acts and emoji usages while discussing COVID-related issues.

Although not within the current research scope, we observed that non-task based chit-chatting that deviated from main discussion topics, contributed to a substantial amount of posts in the thread. In his research on the helping processes in online self-help group focusing on disability issues, Finn (1999) suggested that the discussion of everyday life events in online self-help groups could provide normalizing experience to its members and thus carried therapeutic value. Whether this is the case within the LIHKG thread would require further studies. Swearing and sex chat were also found to be ubiquitous within our samples. This observation resonates Jacobs et al.’s (2022) identification of the LIHKG forum as an embodiment of ‘lad culture’ and share similarities to western manosphere. The potential of these issues to serve as socialization processes among LIHKG members, help them create a sense of community and establishment of LIHKG subculture are worth further investigation on.

The strict social-distancing and large-scale quarantine measures implemented by the Hong Kong government to combat the fifth-wave COVID-19 pandemic has resulted in the social media and online social networks to becoming essential sources of information and socialization in times of fear and uncertainties. They are, however, also potentially serve as a fertile ground for misinformation and disinformation which can adversely impact healthy behaviours, including lesser adhesion to safety rules, lessening risk perception and preventive practices, refusal of expert information and hostility toward vaccines (Scardigno et al., 2023) during the pandemic. Studies on the constructions of misinformation and disinformation related to COVID-19 on LIHKG forum and how they impact users’ health perceptions and behaviours would shed light on our understanding of the impact of infodemic amid a global health crisis.

## Data availability statement

The datasets presented in this study can be found in online repositories. The names of the repository/repositories and accession number(s) can be found at: https://lihkg.com/thread/2905207/page/1.

## Ethics statement

Ethical review and approval was not required for the study on human participants in accordance with the local legislation and institutional requirements. Written informed consent for participation was not required for this study in accordance with the national legislation and the institutional requirements.

## Author contributions

CY was responsible for conceptualization, data acquisition, qualitative and quantitative analysis, and writing of the manuscript. DT and YJ were responsible for qualitative and quantitative analysis. XY was responsible for data acquisition. All authors reviewed and approved the final manuscript.

## Funding

This work was supported by the Distinguished Postdoctoral Fellowship Scheme, Hong Kong Polytechnic University (Project ID: P0035188).

## Conflict of interest

The authors declare that the research was conducted in the absence of any commercial or financial relationships that could be construed as a potential conflict of interest.

## Publisher’s note

All claims expressed in this article are solely those of the authors and do not necessarily represent those of their affiliated organizations, or those of the publisher, the editors and the reviewers. Any product that may be evaluated in this article, or claim that may be made by its manufacturer, is not guaranteed or endorsed by the publisher.

## Footnotes

^1^ https://lihkg.com/robots.txt
